# Supplementary material for: Analysis of Surface Properties of Nickel Alloy Elements Exposed to Impulse Shot Peening with the Use of Positron Annihilation
Source: Materials (Basel). 2021 Nov 30;14(23):7328. doi: 10.3390/ma14237328 (PMC8658380; doi:10.3390/ma14237328)
Supplement: Supplementary file 1 [file materials-14-07328-s001.zip › materials-1448457-supplementary.pdf]

materials

MDPI

Supplementary Material

Analysis of Surface Properties of Nickel Alloy Elements Exposed to Impulse Shot Peening with the Use of Positron Annihilation

Statistical analysis of results

One-way analysis of variance ANOVA (significance level α = 0.05) was employed to assess the significance of the effect of the technological shot peening parameters on the Sa and Sz roughness parameters, relative increase in microhardness ΔHV0.05 (increase in microhardness caused by impulse shot peening compared to the post-milling value), and the mean positron lifetime τ_{mean}. The analyzed variables had a normal distribution confirmed by the Shapiro-Wilk test, and their variances were homogeneous, as verified with the Levene test. The ANOVA analysis of variance could not be performed only for the mean positron lifetime τ_{mean} as a function of shot peening density *j*. This was associated with the non-normal distribution of the values of the dependent variable τ_{mean}.

Table S1 shows the results of ANOVA analysis of the roughness parameters Sa and Sz (the most frequently analyzed parameters in engineering practice).

Table S1. ANOVA analysis of variance for surface roughness parameters Sa and Sz in the applied conditions of impulse shot peening of samples made of the Inconel 718 nickel alloy, where: DF - number of the degrees of freedom, SS - sum of squares between groups, MS - mean sum of squares between groups, F – value of the test statistic, p - probability level.

	Sa				
	**DF**	**SS**	**MS**	**F**	**p**
**E**	5	12.29	2.457	441.83	0.0000
**j**	3	8.69	2.899	506.46	0.0000
**D**	3	20.58	6.861	788.62	0.0000
	**Sz**				
**E**	5	1496.82	299.363	358.81	0.0000
**j**	3	522.63	174.24	366.04	0.0000
**D**	3	1576.21	525.41	503.04	0.0000

Table S2 and S3 show the ANOVA results for the relative increase in microhardness ΔHV0.05 and the mean positron lifetime τ_{mean}, respectively. The analysis revealed that the technological parameters had a significant effect on the values of the 3D roughness parameters, relative microhardness increase, and τ_{mean}. The probability level *p* for all the shot peening conditions is greater than the adopted significance level (α = 0.05), and the $F_{(5;54)}$, $F_{(5;28)}$, $F_{(5;24)}$, $F_{(3;36)}$,$F_{(3;20)}$, and $F_{(3;16)}$ values are higher than F_{α}.

Table S2. ANOVA analysis of variance for the mean positron lifetime τ_{mean} in the applied conditions of impulse shot peening of samples made of the Inconel 718 nickel alloy, where: DF - number of the degrees of freedom, SS - sum of squares groups, F – value of the test statistic, p - probability level.

	τ_{mean}				
	**DF**	**SS**	**MS**	**F**	**p**
**E**	5	263.00	52.60	395.00	0.0000
**j**	3	-	-	-	-
**D**	3	97.50	32.50	285.00	0.0000

Table S3. ANOVA analysis of variance for the relative increase in microhardness Δ HV 0.05 in the applied conditions of impulse shot peening of samples made of the Inconel 718 nickel alloy, where: DF - number of the degrees of freedom, SS - sum of squares between groups, MS - mean sum of squares between groups, F – value of the test statistic, p - probability level.

	ΔHV 0.05				
	**DF**	**SS**	**MS**	**F**	**p**
**E**	5	19747.60	3949.50	66.39	0.0000
**j**	3	3700.39	1233.46	22.7341	0.0000
**D**	3	8229.90	2743.30	48.208	0.0000

The results of the ANOVA analysis of variance demonstrated statistically significant differences in the mean values of the roughness parameters Sa and Sz, the mean increase in microhardness ΔHV 0.05, and the mean positron lifetime τ_{mean} between the analyzed groups. The post-hoc test (Tukey test) was used to check which of the compared groups differed statistically significantly.

Tables S4 and S5 show the results of the post-hoc Tukey test for the dependent variables (roughness parameter Sa and Sz).

Table S4. Comparative analysis of the significance of differences (post-hoc Tukey test) between the mean values of the Sa roughness parameter after the impulse shot peening treatment with the use of different parameters. The red color indicates the level of probability for which there are no statistically significant differences.

**Sa**						
	**E**					
**E**	20	40	60	120	180	240
**20**		0.00013	0.00013	0.00013	0.00013	0.00013
**40**	0.00013		0.03866	0.00014	0.00013	0.00013
**60**	0.00013	0.03866		**0.11642**	0.00013	0.00013
**120**	0.00013	0.00014	**0.11642**			0.00013
**180**	0.00013	0.00013	0.00013	0.00013	0.00013	0.00013
**240**	0.00013	0.00013	0.00013	0.00013	0.00013	

	**j**				**d**			
**j**	44	25	11	6	**d**	3.95	6.00	10.00	12.45
**44**		**0.38754**	0.00016	0.00016	**3.95**		0.00016	0.00016	0.00016
**25**	**0.38754**		0.00016	0.00016	**6.00**	0.00016		0.00016	0.00016
**11**	0.00016	0.00016		0.00016	**10.00**	0.00016	0.00016		0.00016
**6**	0.00016	0.00016	0.00016		**12.45**	0.00016	0.00016	0.00016	

Table S5. Comparative analysis of the significance of differences (post-hoc Tukey test) between the mean values of the Sz roughness parameter after the impulse shot peening treatment with the use of different parameters. The red color indicates the level of probability for which there are no statistically significant differences.

**Sz**						
	**E**					
**E**	20	40	60	120	180	240
**20**		0.00014	0.00014	0.00014	0.00014	0.00014
**40**	0.00014		0.00014	0.00014	0.00014	0.00014
**60**	0.00014	0.00014		**0.18366**	0.00014	0.00014
**120**	0.00014	0.00014	**0.18366**		0.00049	0.00014
**180**	0.00014	0.00014	0.00014	0.00049		0.00014
**240**	0.00014	0.00014	0.00014	0.00014	0.00014	

	**j**				**d**				
**j**	44	25	11	6	**d**	3.95	6.00	10.00	12.45
**44**		0.00016	0.00016	0.00016	**3.95**		0.00016	0.00016	0.00016
**25**	0.00016		0.00022	0.00016	**6.00**	0.00016		0.00016	0.00016
**11**	0.00016	0.00022		0.00016	**10.00**	0.00016	0.00016		0.00016
**6**	0.00016	0.00016	0.00016		**12.45**	0.00016	0.00016	0.00016	

Tables S6 and S7 present the results for the ΔHV 0.05 variable and the dependent τ_{mean} variable, respectively. The analysis demonstrated that the change in the impact energy from *E* = 60 mJ to *E* = 120 mJ did not have a statistically significant effect on the Sa and Sz values. Similarly, there were no statistically significant differences in the Sa roughness parameter values obtained at the shot peening density of *j* = 44 mm^{-2} and *j* = 25 mm^{-2}. The statistical analysis showed statistically significant differences in the values of the Sa and Sz parameters between all diameters of the peening ball *d*.

Table S6. Comparative analysis of the significance of differences (post-hoc Tukey test) between the mean values of the increase in microhardness after the impulse shot peening treatment with the use of different parameters. The red color indicates the level of probability for which there are no statistically significant differences.

ΔHV 0.05						
			E			
**E**	20	40	60	120	180	240
**20**		0.00025	0.00014	0.00014	0.00014	0.00014
**40**	0.00025		0.00964	0.00014	0.00014	0.00014
**60**	0.00014	0.00964		0.07554	0.00099	0.00023
**120**	0.00014	0.00014	0.07554		**0.42485**	**0.10563**
**180**	0.00014	0.00014	0.00099	**0.42485**		**0.95929**
**240**	0.00014	0.00014	0.00023	**0.10563**	**0.95929**	

		j					d		
**j**	44	25	11	6	**d**	3.95	6.00	10.00	12.45
**44**		0.02487	0.00049	0.00019	**3.95**		**0.10772**	0.00023	0.00018
**25**	0.02487		**0.18217**	0.00127	**6.00**	**0.10772**		0.00811	0.00019
**11**	0.00049	**0.18217**		**0.08156**	**10.00**	0.00023	0.00811		0.00078
**6**	0.00019	0.00127	**0.08156**		**12.45**	0.00018	0.00019	0.00078	

Table S7. Comparative analysis of the significance of differences (post-hoc Tukey test) between the mean values of mean positron lifetimes τ_{mean} after the impulse shot peening treatment with the use of different parameters. The red color indicates the level of probability for which there are no statistically significant differences.

τ_{mean}						
			E			
**E**	**20**	**40**	**60**	**120**	180	240
**20**		0.00013	0.00013	0.00013	0.00013	0.00013
**40**	0.00013		0.00013	0.00013	0.00013	0.00013
**60**	0.00013	0.00013		0.00087	0.00020	**0.66960**
**120**	0.00013	0.00013	0.00087		**0, 95452**	0,03398
**180**	0.00013	0.00013	0.00020	**0,95452**		0,00424
**240**	0.00013	0.00013	**0.66960**	0,03398	0,00424	

		d		
**d**	3.95	6.00	10.00	12.45
**3.95**		0.00041	0.02970	0.00017
**6.00**	0.00041		**0.18245**	0.00017
**10.00**	0.02970	**0.18245**		0.00017
**12.45**	0.00175	0.00017	0.00017	

The analysis of the influence of technological parameters on the increase in microhardness ΔHV 0.05 (Table S6) showed no statistically significant effect of changes in the impact energy from *E* = 120 mJ to *E* = 180 mJ, from *E* = 120 mJ to *E* = 240 mJ, and from *E* = 180 mJ to *E* = 240 mJ on the analyzed dependent variable. There were no differences in the ΔHV 0.05 values between the shot peening density of *j* = 6 mm^{-2} and *j* = 11 mm^{-2} and between *j* = 11 mm^{-2} and *j* = 25 mm^{-2}. The change in the ball diameter *d* from 3.95 mm to 6.00 mm did not exert a significant effect on ΔHV 0.05.

The analysis of the results presented in Table S7 revealed no statistically significant differences in the mean positron lifetime τ_{mean} upon the changes in impact energy from 60 mJ to 240 mJ and from 120 mJ to 180 mJ. Additionally, no statistically significant differ-

ences were found in the values of the mean positron lifetime between treatments with the different ball diameters of 6 mm or 10 mm.

The ANOVA analysis of variance for the surface roughness parameters, relative increase in microhardness ΔHV 0.05, and mean positron lifetime τ_{mean} revealed a significant effect of the impulse shot-peening conditions on the results. Noteworthy is the absence of significant effects on the analyzed variables in the case of most of the same peening conditions. This may indicate that there is a correlation between the values of microhardness, surface roughness, and mean positron lifetimes.

Surface non-homogeneity

In the case of the use of relatively high energies (E = 180 mJ) and small balls (d = 6.00 mm), the dispersion of the mean lifetime significantly exceeded the statistical uncertainty when the measurements were conducted in randomly selected locations of the positron source (Figure S1). This indicates non-homogeneity of the surface and requires systematic surface scanning and averaging the results in order to obtain reliable measurement results. Hence, a specialized stand is required to control the location of the positron source relative to the sample.

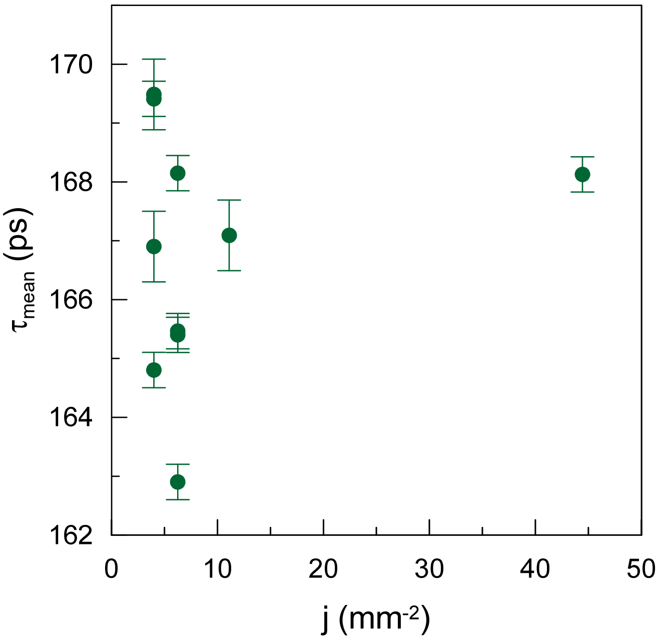

Figure S1. Dependence of the mean positron lifetime τ_{mean} on the shot peening density j (E = 180 mJ, d = 6.00 mm).
